# Cytokines and Growth Factors Expressed by Human Cutaneous Melanoma

**DOI:** 10.3390/cancers2020794

**Published:** 2010-05-07

**Authors:** Elias G. Elias, Joanne H. Hasskamp, Bhuvnesh K. Sharma

**Affiliations:** Maryland Melanoma Center, Weinberg Cancer Institute, Franklin Square Hospital Center, Baltimore, MD, USA; E-Mails: joanne.hasskamp@medstar.net (J.H.H.); bhuvnesh.sharma@medstar.net (B.K.S.)

**Keywords:** melanoma, cytokines, growth factors

## Abstract

Cytokines and growth factors have biologic effects that could stimulate tumor growth, invasion and angiogenesis. The incidence of 24 factors was investigated in 25 cultured human melanoma cell lines and in 62 fixed tissues at different stages of the disease. Over 80% of the human melanoma cell lines expressed TGF-β, IL-8, IL-6, VEGF, PDGF-AA and OPN. Significantly higher TGF-β, IGF-1 and IL-15 were determined in primary lesions compared to distant metastases by immunohistochemistry. Illustrating the complexity of the milieu of the tumor microenvironment, some of these factors may have to be considered in targeted therapy.

## 1. Introduction

Many studies have implicated cytokines and growth factors in tumor growth, survival and migration in various human cancers. Melanoma has multifactorial etiology and its genetic and immunological background has not yet been clearly elucidated. *In vitro* studies seem to indicate that cultured melanoma cell lines produce excessive levels of several cytokines and growth factors, which have various biological effects. Some of them function as autocrine factors and/or paracrine factors and are capable of stimulating tumor growth, invasion and angiogenesis [1,2]. Others function as adhesion molecules [3,4,5], and anti-apoptotic proteins [6,7]. It is assumed that modifying the milieu of factors within the tumor might alter the outcome of the host response to cancer therapy. Therefore, the study of these factors could improve the understanding of the biology of melanoma and may help in directing targeted therapy. The present study was designed to assess secreted factors in the supernatant of cultured human melanoma cell lines established from lymph nodes (LN) and distant metastases. In addition, *in situ* overexpression of these factors was studied in melanoma tissues from various stages of the disease utilizing immunohistochemistry (IHC). We focused our IHC studies on overexpression of cytokines and growth factors in primary and metastatic melanoma in order to delineate potent potential therapeutic targets. Our IHC study excluded stromal expression of these factors to define overexpression of factors only by the melanoma cells in the progression of the disease.

## 2. Results

### 2.1. Cultured Human Melanoma Cell Lines

Eighteen of 24 factors were detected by ELISA at different incidences and levels in the spent medium from human melanoma cell lines after seven days of culture. Each cell line secreted 5-12 factors. Six factors were not identified in any of the spent medium samples. All cell lines secreted IL-8 and TGF-β, and 95% secreted VEGF-A as can be seen in Table 1 and Table 2.

**Table 1:** Incidence of cytokines detected in the spent medium of human melanoma cell lines after seven days of culture.

Cytokine	ELISA sensitivity (pg/mL)	Number of cell lines tested	Percentage of cell lines secreting cytokine
IL-8	31	16	100
IL-6	10	15	87
OPN	312	22	82
TNF-α	16	15	53
IL-10	15	17	47
GM-CSF	15	14	43
IL-1 α	13	15	40
IFN -α	10	16	19
IL-15	4	15	7
IL-4	16	17	0
IL-13	26	17	0
IL-17	31	17	0
IL-18	26	17	0
IFN-β	250	17	0

**Table 2:** Incidence of growth factors detected in the spent medium of human melanoma cell lines after seven days of culture.

Growth Factor	ELISA sensitivity (pg/mL)	Number of cell lines tested	Percentage of cell lines secreting growth factor
TGF-β	31	25	100
VEGF	31	22	95
PDGF-AA	31	21	86
PlGF	16	22	59
PDGF-AB	31	23	52
TGF-α	16	21	52
FGF-b	10	22	45
IGF-1	94	21	33
EGF	4	22	9
PDGF-BB	31	25	0

### 2.2. Heterogeneity of Factors

Melanoma cell lines derived from two patients who had recurrent metastases generated data exemplifying the heterogeneity of melanoma. The results from ELISA assays for platelet derived growth factor (PDGF-AA), osteopontin (OPN), and placenta growth factor (PlGF) can be seen in Table 3. It is of interest to note the variation in the results obtained from different metastatic sites of the same patient on the same day.

**Table 3:** Concentrations of factors in the spent medium from melanoma cell lines derived from recurrent metastases in each of two patients are shown. Cultures were normalized for cell number, seeding 3–4 million viable cells per flask. Average concentrations and two times the standard deviation from triplicate experiments are shown. ELISA sensitivity is the same as in Table 1 and Table 2. Heterogeneity inherent in melanoma is reflected in the data.

Patient number	Source of tumor	Date collected	PDGF-AA mean pg/mL (2 SD)	OPN mean pg/mL (2 SD)	PlGF mean pg/mL (2 SD)
**1**	Lymph node	14 April 2005	0	3533 (5801)	0
	Soft tissue	8 June 2005	0	2467 (1617)	95 (139)
	Lymph node	8 June 2005	1245 (1414)	100 (346)	32 (19)
**2**	Lymph node, Deep groin	1 September 2006	4783 (685)	60763 (11127)	4387 (455)
	Lymph node, Superficial groin	1 September 2006	680 (311)	467 (115)	0

### 2.3. Immunohistochemistry Study

**Figure 1:**
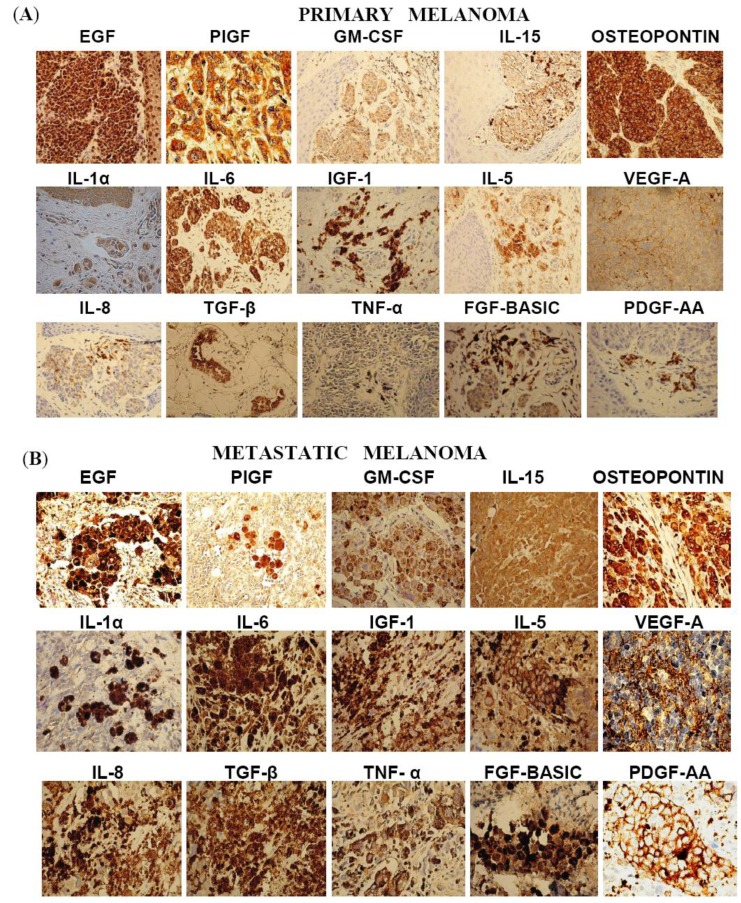
Images of representative IHC staining for factors in primary melanoma (A) and metastatic melanoma (B).

High levels of expression of cytokines and growth factors were evaluated in terms of intensity of staining as well as the percentage of immunostained tumor cells, *i.e*., extent. The natural history of the melanoma was considered by assessing lesions from primary sites, local recurrences, intransit metastases, lymph node metastases and distant metastases. There was extreme variation in the expression of cytokines and growth factors among different stages of the disease. For statistical analysis of the two extremes, Fisher’s Exact test was applied to the IHC data from the primary lesions and the distant metastases. Table 4 shows the incidence of factors with high intensity in the IHC staining, while Table 5 represents the results by extent of IHC staining. Figure 1 shows images of representative IHC staining in primary melanoma and metastatic melanoma for the factors tabulated in Table 4 and Table 5. There was overexpression of EGF in almost all tissues tested. Ten factors had higher incidence of overexpression in primary lesions compared to metastatic lesions by intensity of immunostaining, but only TGF-β (p < 0.001) reached statistical significance. Comparisons of the percentages of immunostained cells in primary lesions versus distant metastases showed statistically significant differences for IGF-1 (p < 0.01) and IL-15 (p < 0.05). 

**Table 4:** Percent of melanoma cases overexpressing the cytokines/growth factors evaluated by the intensity of immunohistochemical staining.

Cytokines/Growth Factors	Primary Melanoma (n = 12)	Distant Metastasis (n = 19)	Fisher’s Exact Test P-value
EGF	12 (100%)	14 (74%)	NS
PlGF	6 (50%)	7 (37%)	NS
GM-CSF	4 (33%)	2 (11%)	NS
IL-15	5 (42%)	3 (16%)	NS
Osteopontin	12 (100%)	15 (79%)	NS
IL-1α	8 (66%)	15 (79%)	NS
IL-6	7 (58%)	12 (63%)	NS
IGF-1	5 (42%)	8 (42%)	NS
IL-5	7 (58%)	14 (74%)	NS
VEGF-A	2 (17%)	4 (21%)	NS
IL-8	7 (58%)	4 (21%)	NS
TGF-β	**11 (92%)**	**3 (16%)**	**<0.001**
TNF-α	2 (17%)	8 (42%)	NS
FGF-Basic	8 (67%)	9 (47%)	NS
PDGF-AA	1 (8%)	8 (42%)	NS

**Table 5:** Percent of melanoma cases over-expressing the cytokines/growth factors evaluated by the extent of immunohistochemical stained tumor cells.

Cytokines/Growth Factors	Primary Melanoma (n = 12)	Distant Metastasis (n = 19)	Fisher’s Exact Test P-value
EGF	12 (100%)	19 (100%)	NS
PlGF	11 (91%)	18 (94%)	NS
GM-CSF	9 (75%)	12 (63%)	NS
IL-15	**9 (75%)**	**5 (26%)**	**<0.05**
Osteopontin	9 (75%)	9 (47%)	NS
IL-1α	8 (66%)	11 (57%)	NS
IL-6	8 (66%)	8 (42%)	NS
IGF-1	**7 (58%)**	**1 (5%)**	**<0.01**
IL-5	7 (58%)	9 (47%)	NS
VEGF-A	3 (25%)	3 (15%)	NS
IL-8	4 (33%)	10 (52%)	NS
TGF-β	1 (8%)	5 (26%)	NS
TNF-α	10 (83%)	11 (57%)	NS
FGF-Basic	**10 (83%)**	**8 (42%)**	**<0.05**
PDGF-AA	6 (50%)	8 (42%)	NS

## 3. Discussion

### 3.1. Duality of Effects

Clinical trials in the management of melanoma have shown that some cytokines are beneficial and have anti-neoplastic effect such as interferon α-2b and interleukin-2. The present *in vitro* studies utilizing melanoma cultured cell lines revealed that melanoma cells express a variety of cytokines and growth factors, and some of them may stimulate tumor angiogenesis, growth and invasion [2]. Some cytokines/growth factors may function as autocrine factors (to stimulate tumor growth) while others as paracrine factors (to stimulate tumor invasion). Furthermore, some of them can switch from an autocrine to a paracrine function. Cytokines, soluble proteins or glycoproteins present in the host stroma and various immune cells [8], can be excessively produced in response to antigenic stimuli. Cytokines and growth factors have an impact on many critical biological processes, with diverse effects ranging from acute regulation of gene expression and cell proliferation to promotion of chronic inflammation [9]. These factors can be involved in the activation of effector mechanisms that limit tumor growth [10] for effects beneficial to patients. Contrary to this, they contribute to inflammation, transformation, tumor growth and invasion [11].

### 3.2. Cytokines

#### 3.2.1. Interleukins

Interleukins detected in the culture spent medium were IL-8, IL-6, IL-10, IL-1α and IL-15. All of the melanoma cell lines secreted IL-8. IL-8 is an angiogenic factor expressed at high levels in a variety of vascular cancers [12]. IL-8 binds to chemokine receptors CXCR-1 and CXCR-2, which also has additional ligands [13]. CXCR1 and CXCR2 are expressed by melanoma and are involved in melanoma proliferation and metastasis [14,15,16]. Interleukin-6 (IL-6) is a pleiotropic cytokine produced by T cells, B cells, monocytes, fibroblasts, endothelial cells and several types of tumor cells. It is a differentiation factor for T cells, B cells and macrophages [17]. Melanoma patients with high serum IL-6 have a shorter survival and a tendency to be resistant to IL-2 therapy [18]. IL-10 is closely related to interferon α and γ [19]. It has complex biological functions. It may act as a tumor growth factor. It was identified in the serum of patients with metastatic melanoma [20]. IL-1α is a pro-inflammatory cytokine that initiates an immune response to apoptotic cells [21]. Its production has been reported in melanoma [22]. IL-15 is produced by monocytes, macrophages, dendritic cells and bone marrow stromal cells. It acts primarily on lymphocytes and affects memory CD8+ T cells [23]. Its deregulation can lead to the development of cutaneous lymphoma, lymphocytic leukemia and multiple myeloma. IL-15 is structurally similar to IL-2 and signals through the IL-2 receptor [24]. *Ex vivo* expansion of tumor specific lymphocytes using IL-15 and IL-21 produced fewer Tregs and more cytolytic cells against melanoma cell lines [25]. However, IL-15 has also been shown to stimulate a melanoma cell line that expressed the IL-2R [26].

Conversely, the interleukins IL-4, IL-13, IL-17 and IL-18 were not detected in the spent medium from our human melanoma cell lines. These cytokines have immune modulating functions. IL-4 and IL-13 are produced by Th2 cells polarizing a humoral immune response. IL-17 is produced by Th17 effector T helper cells. Its proinflammatory function has dual effects on cancer, promoting antitumor cytotoxic T responses but also promoting angiogenesis and metastasis [27]. Increased Th17 cells have been reported in the blood of melanoma patients with autoimmune toxicities to the anti-CTLA4 antibody tremelimumab [28]. IL-18 induces IFN-γ production by T and NK cells. IL-18 expression has been reported in some cutaneous melanomas [29]. However, IL-18 expression was found to be reduced in ultraviolet radiation-induced melanoma [30]. The unexpected result of no measurable IL-18 in our culture spent medium suggests that melanoma from our patient population includes ultraviolet radiation-induced melanoma.

#### 3.2.2. Other Cytokines

Other cytokines detected in the spent medium were osteopontin (OPN), tumor necrosis factor-α (TNF-α), interferon-alpha (IFN-α) and granulocyte-macrophage colony stimulating factor (GM-CSF). OPN is an extracellular structural protein which is expressed in a wide range of immune cells. It has multiple functions involving cell adhesion, chemotaxis, invasion, anti-apoptosis and regulation of cell signaling in almost all malignancies. The elevated serum level is associated with poor prognosis [31]. TNF-α is considered a factor in cancer cachexia. It is produced by macrophages, monocytes, fibroblasts, keratinocytes and other cells. It is involved in systemic inflammation that stimulates the acute phase reaction. TNF-α is the ligand of epidermal growth factor receptor. It may function in autocrine or paracrine fashions and contributes to tumor survival through anti-apoptotic gene expression [32]. On the other hand, it is a pro-apoptotic mediator when combined with interferon. TNF-α is being used clinically in combination with melphalan in limb perfusion in the management of melanoma of the extremity [33]. IFN-α is an angiogenic inhibitor with anti-proliferative as well as pro-apoptotic effect, especially if combined with TNF [34,35]. Isolated limb perfusion with TNF and melphalan followed by low-dose IFN-α systemic therapy improved the progression-free survival in a pilot study of melanoma patients with in-transit metastases [36]. Further study is needed to evaluate the potential beneficial effect of combination therapy with TNF and IFN-α. IFN-β is similar to interferon-alpha but with less activity. GM-CSF has anti-tumor effects by generating antigen presenting cells (APC) that are effective in processing dying cells and inducing cellular and humoral immunity [37]. It is being utilized in several aspects of melanoma management. 

### 3.3. The Growth Factors

Transforming growth factor-beta 1 (TGF-β1) was detected in the spent medium of all 25 of the melanoma cell lines and also was statistically significant in the IHC intensity comparison of primary lesions versus metastases. TGF-β1 is initially a tumor suppressor, but once the tumor is established, its signaling can enhance tumor progression. Some tumors can escape the suppressive effect through mutation of receptor I/II but this mechanism of resistance has not been identified in melanoma [38]. 

Vascular endothelial growth factor-A (VEGF-A) is secreted by normal and tumor cells, with a significant portion bound to the endothelial surface and extra-cellular matrix [39]. It has nine isoforms and six homologs with a variety of receptors. VEGF is an angiogenic factor in melanoma that is targeted by drugs such as bevacizumab. Early clinical trials are investigating the treatment of melanoma with bevacizumab in monotherapy (NCI protocol NSD-11933) as well as in various combinations (NCI protocol 08-142, NCI Protocol POHA-0603, NCI protocol NCCTG-N0879 and others).

Platelet derived growth factors PDGF-AA and PDGF-AB are members of a family of isoforms that stimulate the growth, survival and motility of fibroblasts, smooth muscle cells and other cell types. Beside their mitogenic activity, they activate fibroblasts to produce insulin-like growth factor-1 (IGF-1), which in turn stimulates the growth of some malignant cells that express its receptor [40]. 

IGF-1 (insulin-like growth factor 1) is a potent growth and survival factor secreted in 33% of the cell lines. It promotes proliferation and cell survival through activation of MEK/ERK and P13-k/Akt signaling cascades in the cell [41]. IGF-1 signaling through its receptor increases the expression of anti-apoptosis proteins Bcl-2, Bcl-xl and survivin [42]. IGF-1 has been identified as a target for targeted therapy. Inhibitors and antagonists of the IGF-1 receptor such as cixutumumab, BMS-754807 and OSI-906 are in early clinical trials but have not yet been tested in melanoma. 

Placenta growth factor (PlGF) was secreted in 59% of the cultured cell lines and seen in the majority of the melanoma tissues by extent of IHC staining in Table 5. PlGF is a member of the VEGF family of proteins. The function of PlGF on melanoma angiogenesis and metastasis is an area of active research [43]. 

TGF-α contributes to changes in gene expression resulting in uncontrolled growth and avoidance of apoptosis [44,45]. 

FGF-b (fibroblast growth factor-basic) stimulates angiogenesis and cell growth of cells from mesodermal and muco-ectodermal origin including endothelial cells and fibroblasts. It regulates proliferation, differentiation and migration [46]. 

EGF (epidermal growth factor) is an angiogenic factor and very potent mitogen for endothelial cells [47,48]. It has been claimed to have paracrine function in several malignancies. Elevated levels of the EGF receptor (EGFR) have correlated to poor prognosis in head and neck, ovarian, cervical, bladder and oesophageal cancers [49] but substantial similar data for melanoma is not yet available. Increased copy number for EGFR and chromosome 7 alterations have been reported in primary cutaneous melanoma to be associated with poor prognosis [50]. Preclinical studies in a mouse xenotransplantation model of melanoma showed synergistic activity of the EGFR inhibitor erlotinib in combination with bevacizumab resulting in reduced tumor volume [51]. Further investigation is needed of the application of EGFR targeted therapies to melanoma.

PDGF-BB was not secreted by any of our cultured cell lines. However, it was evaluated because of the co-expression of PDGF-B chain and its receptor in soft tissue sarcoma [52]. PDGFB deficiency may cause anomalies in placental blood vessels [53].

### 3.4. Comparison of Factors from Cell Lines and Tissues

There were no concordance between the levels of secreted factors of the cultured cell lines and their IHC expression in paraffinized tissues. While cultured cell lines consisted of actively dividing cells in an artificial environment, IHC expressed the results in fixed tissue from a snapshot in time. The cultured cell lines were established from lymph node and distant metastases, whereas IHC expressed factors presented at different stages of the disease. 

### 3.5. Clinical Considerations

While it was expected that some cytokines/growth factors would increase with the progression of the disease, the opposite results were obtained. The results of IHC revealed that there were no significant differences among local recurrences, intransit, regional lymph node or distant metastases. There were more factors overexpressed in primary lesions (10 by IHC intensity, 13 by IHC extent) than in metastases (seven by IHC intensity, three by IHC extent). This may indicate that these factors may be required in the early stages of the disease and to a lesser extent in later stages.

The results from patients with recurrent metastases at different sites suggest that various clones of tumor cells can be present at various sites, in the same patient and on the same day. This may have an impact on the therapeutic approaches, especially autologous vaccines, and explain the reason for initial failure or initial response then failure to therapy. Repeated biopsies of recurrences and metastases may be required to assess the status of various cytokines/growth factors in order to redirect therapy.

GM-CSF is of special interest as it is being administered clinically as adjuvant therapy to induce APC for melanoma vaccines. Dendritic cells and macrophages are effective in capturing dying tumor cells and inducing cellular and humeral immunity [37]. On the other hand, it has been reported that high-dose GM-CSF given with vaccines can be immunosuppressive [54,55]. Therefore, its presence in melanoma tissue may indicate local immunosuppression by tumor cells, or it could induce dendritic cells and macrophages to clean the debris of dead tumor cells.

## 4. Experimental Section

### 4.1. Establishment and Characterization of Human Melanoma Cell Lines

Twenty-five human melanoma cell lines were established from metastatic tumors of consenting patients. The majority of the cell lines [17] were derived from lymph node metastases. The remaining cell lines were obtained from metastases in small bowel [1], ovary [1], lung [1], soft tissues of limbs [3] and subcutaneous tissue [1]. The collection of melanoma cell lines included two cell lines from a given patient derived at different times as well as two cell lines from each of three patients derived from different tumors removed from different sites on the same day.

Single cell suspensions were prepared from freshly resected specimens by mechanical mincing. No enzymatic dissociation was used. Viable tumor cells were cultured in Iscove’s medium supplemented with 10% fetal bovine serum (FBS) and antibiotics. After overnight incubation at 37 °C with 5% CO_2_, the floating debris was discarded and fresh complete medium was added. The cultures were fed 2–3 times per week, replacing about half of the spent medium. Melanoma cell lines were split when near confluence and subcultured at 4 × 10^4^ viable cells per cm^2^ surface area in polystyrene culture flasks. Cultures were shown to be free of mycoplasma contamination using the MycoProbe^TM^ mycoplasma detection kit (R&D Systems, Minneapolis, MN, USA). To insure that each cultured cell line consisted of melanoma cells, each cell line was stained for melanoma-specific antigens MART-1, gp100, TRP75, or melanoma-associated chondroitin sulfate proteoglycan (MCSP) and analyzed by flow cytometry. At least one of these four melanoma antigens was observed in order to include the cell line in the study. All cell lines were early passages, ranging from 2 to 36 passes. 

Expression of melanoma antigens MART-1, gp100 and TRP75 was determined by permeabilizing with Cytofix/Cytoperm (BD Biosciences) and Perm/Wash solution (BD Biosciences), staining 30 minutes at room temperature protected from light and washing with Perm/Wash solution. Surface staining for MCSP included the viability stain 7AAD. Surface antigens were stained for 15 minutes at room temperature protected from light and washed with 0.5% BSA-PBS-0.1% sodium azide solution. Samples were centrifuged at 450× g for 5 minutes, stored in 1% paraformaldehyde-PBS pH7.25 and run through the FACSCalibur flow cytometer (BD Biosciences) within 24 hours of fixation. Isotype controls were performed for each test. Antibodies used to stain for melanoma antigens are listed in Table 6.

After seeding 3–4 million viable cells, cultures were fed 2–3 times per week by measuring the amount of spent medium that was removed and replacing it with fresh medium. Samples of the spent medium were collected after 7 days of culture to allow for approximately 2–3 doublings and concentrations of factors to accumulate. Commercially available ELISA kits (R&D Systems, Minneapolis, MN; Pierce Biotechnology, Rockford, IL; PBL Biomedical Laboratories, Piscataway, NJ; Medical and Biological Laboratories, Nagoya, Japan) were used to measure the factors secreted by the melanoma cell lines. The lowest standard on the standard curve was considered the ELISA sensitivity. Complete Iscove’s-10% FBS was used in the standard curves and as the blank in the ELISA assays to control for any potential cross reactivity from bovine cytokines/growth factors in the complete medium. Triplicate experiments were performed for each factor. The spent media were evaluated for the presence and levels of 24 factors in 17 to 25 cell lines. This study does not attempt to evaluate the functional level of factors but rather evaluates the complexity of the mixture of factors secreted by melanoma.

**Table 6:** Antibodies used for staining, their characteristics and sources.

Antibody	Clone	Intracellular/Surface	Source
Anti-MART-1	M2-7C10	Intracellular	Lab Vision NeoMarkers MS-612P
Anti-gp100	HMB45	Intracellular	Invitrogen 18-2050
Anti-TRP75	TA99	Intracellular	Lab Vision NeoMarkets MS1634P
MCSP-APC	EP-1	Surface	Miltenyi Biotec 130-091-252

### 4.2. Immunohistochemistry Studies

Five-micron thick sections obtained from 62 fixed melanoma tissues (paraffin blocks) were stained for 17 factors by IHC. The specimens represented 12 primary lesions, 10 local recurrences, 6 intransit metastases, 15 regional lymph node metastases and 19 distant metastases. Each case was individually stained for each antibody utilizing commercially available monoclonal/polyclonal antibodies (Table 7) and biotin-free Bond polymer refine detection kit (Leica Microsystems) using the Bond-Max automated IHC system (Leica). The polymer refine detection system was used for high sensitivity probing and amplification of immunostaining. Endogenous melanin was of a much lower intensity when present and caused no technical difficulty in evaluation of the overexpression of factors. Positive controls based on literature as well as recommendations of antibody vendors were used to establish the optimal dilution of the antibody and to establish antigen retrieval conditions. The pattern of expression of each factor was evaluated under a Zeiss Axioscope microscope with a semi-quantitative scoring system as: 0 = negative (no staining), 1+ = weak expression, 2+ = moderate expression, 3+ = overexpression by intensity of staining of tumor cells. On the other hand, the extent of staining of the tumor cells was scored as: 0 = negative, 1 = 1%–33% of the cells were stained, 2 = 33%–66%, 3 = >66% of the cells stained (overexpression) based on cell count in three fields of 400× magnification. Digital images were obtained and kept on file. 

**Table 7:** Details of the antibodies used for IHC of cytokines and growth factors.

Antibody	Type of antibody	Catalog #	Vendor
IL-1α	Monoclonal	MAB 200	R&D Systems, Minneapolis, MN
IL-5	Monoclonal	MAB-605	R&D Systems, Minneapolis, MN
IL-6	Monoclonal	NCL-IL-6	Nova Castra, New Castle upon Tyne, UK
IL-8	Polyclonal	AHC-0881	Biosource, Camarillo, CA
IL-15	Monoclonal	MAB 647	R&D Systems, Minneapolis, MN
GM-CSF	Monoclonal	MAB 215	R&D Systems, Minneapolis, MN
VEGF-A	Monoclonal	MAB-293	R&D Systems, Minneapolis, MN
PDGF-AA	Polyclonal	AF-221NA	R&D Systems, Minneapolis, MN
Osteopontin	Monoclonal	NCL-OPNTIN	Nova Castra, New Castle Upon Tyne, UK
IGF-1	Polyclonal	AF-291NA	R&D Systems, Minneapolis, MN
PlGF	Monoclonal	P-3863	Sigma, St Louis, MO
TGF-β	Monoclonal	NCL-TGF-B	NovaCastra, New Castle Upon tyne, UK
FGF-basic	Polyclonal	AB-233-NA	R&D Systems, Minneapolis, MN
EGF	Monoclonal	E-2520	Sigma, St Louis, MO
TGF-alpha	Polyclonal	AF-239-NA	R&D Systems, Minneapolis, MN

## 5. Conclusions

A component of the heterogeneity inherent in melanoma is the secretion and expression of various cytokines and growth factors at variable levels. The high incidence of expression of TGF-β, IL-8, IL-6, VEGF, PDGF-AA, OPN, IGF-1 and IL-15 by human melanoma supports consideration of these factors in future planning of therapy. Due to the heterogeneity of melanoma, repeated biopsies of metastases may have to be considered to redirect future therapy as personalized medicine develops. It will be the challenge of personalized medicine to develop appropriate diagnostics for criteria of therapeutic choices.

## Abbreviations

IL: interleukin
TNF-α: tumor necrosis factor-alpha
IFN: interferon
TGF: transforming growth factor
VEGF: vascular endothelial growth factor
PDGF: platelet derived growth factor
PlGF: placenta growth factor
FGF: fibroblast growth factor
IGF: insulin-like growth factor
EGF: epidermal growth factor
OPN: osteopontin
